# Growth Traits and Electrophysiological Responses of *Cardamine violifolia* to Selenium Biofortification Under Various Selenomethionine Levels

**DOI:** 10.3390/plants15172680

**Published:** 2026-08-31

**Authors:** Antong Xia, Jingjing Zhou, Yijun Wang, Sirong Chen, Kun Zhai, Dongshan Xiang

**Affiliations:** 1 Hubei Key Laboratory of Selenium Resources Research and Biological Application, Hubei Minzu University, Enshi 445000, China; 202311437@hbmzu.edu.cn (J.Z.); 202311417@hbmzu.edu.cn (Y.W.); 202314203@hbmzu.edu.cn (S.C.); 1998011@hbmzu.edu.cn (K.Z.); 2 School of Chemistry and Environmental Engineering, Hubei Minzu University, Enshi 445000, China

**Keywords:** hyper-selenophilous plant species, SeMet fortification, electrophysiological nutrient metabolism, real-time monitoring technology, growth traits

## Abstract

Selenomethionine (SeMet) is essential for selenium fortification in the hyper-selenophilous plant, *Cardamine violifolia* (*Cv*). However, there is currently a lack of real-time monitoring techniques to investigate SeMetfortification in *Cv*. In this study, we employed a time-course experiment (0–10 days) under different concentrations of SeMet (0–300 mg/L). Based on the growth characteristics of *Cv*, SeMet supplementation was most effective on day 4 at 100 mg/L. Compared with CK, the fresh weight of the roots, stems, and leaves, as well as the total chlorophyll and total nitrogen content, increased by only 2.7–32.9%. Furthermore, based on electrophysiological water metabolism and nutrient translocation in *Cv* leaves, an electrophysiological indicator, ESR, was used to evaluate selenium biofortification with SeMet. We found that electrophysiological responses are more sensitive than growth traits; the intrinsic capacitance (ICp) of S2 is 173% higher than that of CK, whilst IR, IZ, IX_C_ and IX_L_ are reduced from 44.4% to 73.68%. Intracellular water-holding capacity (IWHC), water transfer rate (WTR), nutrient translocation rate (NTR), and electrophysiological metabolic activity (MA) increased by 144–264%, enhancing SeMet enrichment efficiency. However, when SeMet > 100 mg/L (S3–S6), intracellular water metabolism and active nutrient transport were reduced, leading to the IWHC of S6 being 32.8% lower than CK. Moreover, although the ESR of S6 is higher than CK, the total SeMet transport capacity (STC) of *Cv* is reduced, promoting the efflux of SeMet (ES2) to inhibit high SeMet stress. Correlation analysis indicated that the electrophysiological selenium-enhancing rate (ESR) is significantly positively correlated with *Cv* biomass (R^2^ = 0.89, *p* ≤ 0.05) and leaf area (R^2^ = 0.85, *p* ≤ 0.05), showing that it served as an electrophysiological factor of selenium biofortification. Hence, plant electrophysiological technology enables real-time monitoring of different SeMet biofortification in *Cv*, and ESR can provide a useful reference for assessing selenium biofortification in hyperaccumulators.

## 1. Introduction

Most regions in China suffer from a scarcity of selenium resources, and the population generally has an inadequate selenium intake of 43.3 μg/day, which is less than the recommended daily intake of 60 μg/day. Consequently, plant-based foods are a key strategy for alleviating selenium deficiency in humans [1]. *Cardamine violifolia* (*Cv*), a hyper-selenophilous plant native to Enshi (E 109°04′–109°58′, N 29°50′–30°39′, Hubei province, China), has a selenium accumulation capacity of up to 7816 mg/kg, making it the most potent selenium-accumulating plant around the world [2], with biologically selenium forms, such as selenomethionine (C_5_H_11_NO_2_Se), selenocysteine (C_3_H_7_NO_2_Se), and L-selenomethylselenocysteine (C_4_H_9_NO_2_Se), which offer significant economic and health benefits [3]. To optimize the efficient utilization of bio-organic selenium in *Cv* and enhance its medicinal and dietary value, exogenous selenium fortification is a key method for improving selenium bioavailability in *Cv.* It is also crucial to the sustainable development of the selenium-enriched industry [4].

Selenomethionine (SeMet), a commonly used exogenous selenium fortifier, offers the advantages of high biological activity and high absorption rates in humans; it can be directly absorbed and utilized by the body via the amino acid transport system. It significantly increases the bioavailable selenium content and health benefits of *Cv*, offering considerable selenium supplementation effects [5]. At the same time, SeMet at an appropriate concentration can not only activate the antioxidant enzyme system [6] and alleviate oxidative stress [7], but it can also promote selenium–cadmium (or other heavy metals) antagonism [8], thereby supporting healthy cultivation of *Cv.* By promoting healthy cultivation of *Cv*, the above research has laid a solid foundation for monitoring the effectiveness of selenium fortification in *Cv*. However, there is a lack of research on real-time monitoring techniques for SeMet fortification in *Cv*.

Studies have shown that electrical signals in plants are key indicators of their real-time physiological responses, attributed to capacitive, inductive, and resistive properties of cells, which can convert energy under the growth of plant species [9]. Therefore, scientists believe that electrical signals in plants accompany physiological processes. For example, during H^+^ transport, the current and potential gradients generated by the H^+^-ATPase proton pump, along with ATP synthesis, are used to characterize real-time H^+^ transport dynamics [10]. Since then, scholars have used patch-clamp [11] and microneedle electrodes [12] to measure physiological resistances of plant electrical signals. However, all of the above methods disrupt tissues and cells, making it difficult to reflect the real-time electrical signals of plant species accurately. Consequently, it is vital to refine a real-time electrophysiological monitoring technique and accurately measure dynamic growth traits of plant species.

Compared with other traditional technologies, the electrophysiological monitoring technique is a fast, real-time, and lossless method to record dynamic electrophysiological parameters. Based on non-damaging microelectrodes, it uses LCR testers to measure the real-time capacitance (Cp), resistance (R), and impedance (Z) of leaves under different clamping forces (F). Subsequently, based on the Nernst equation, Gibbs free energy, and thermodynamic principles, we constructed an electrophysiological model to quantify electrophysiological water metabolism, nutrient transport, and energy metabolism [13]. It not only enables plant dynamic metabolism, such as water metabolism and nutrient transport [10], but also adaptability to environmental stresses such as drought [9], low temperature [14], and high Na^+^ stress [15]. Importantly, we made considerable progress in exploring exogenous selenium effects by electrophysiological monitoring techniques. In previous studies, we found that HCO^3−^ cooperates with Se^4+^ to promote electrophysiological water and active nutrients, alleviating cadmium stress in *Cv* [16]. In addition, under non-flooding conditions, silicate (Si^4+^) synergizes with Se^4+^ to promote the active electrophysiological nutrient transport of Yixiangyou 876 rice, improving selenium–cadmium antagonism [16]. Consequently, plant electrophysiological monitoring techniques served as a reliable method to track selenium biofortification traits of *Cv* under various SeMet levels.

In this study, *Cv* was selected as the experimental material. Following the safety threshold for SeMet enhancement in cruciferous plants pecies [17], we set up a time period with various SeMet levels to treat *Cv* seedlings. Based on *Cv*’s growth characteristics, we determined the optimal application time of SeMet. Subsequently, using plant electrophysiological technology, we assessed leaf electrophysiological water metabolism, nutrient transport, and metabolic activities to identify the most suitable SeMet level for *Cv*. Finally, based on dynamic electrophysiological water metabolism and nutrient transport traits, we constructed the electrophysiological selenium enhancement rate (ESR) to provide a new real-time method for precision selenium biofortification of *Cv*. This study provides a reliable electrophysiological evaluation method for *Cardamine violifolia* SeMet adaptation and further facilitates the sustainable development of selenium-biofortified agriculture.

## 2. Materials and Methods

### 2.1. Experimental Materials

In this study, healthy *Cv* seedlings were collected in the industrial park of the Academy of Agricultural Sciences of Enshi Tujia and Miao Autonomous Prefecture (E 109°49′, N 30°32′), stored in foam boxes at low temperature, and transported to the laboratory for backup. Subsequent seedling cultivation and the full set of SeMet treatment experiments were conducted under controlled laboratory conditions. Prior to the application of SeMet, seedlings of uniform age, plant height, leaf number and vigor and free from visible damage were selected and randomly allocated to each treatment group to minimize initial individual variation as far as possible. Throughout the experiment, all treatments were cultivated synchronously, and samples were collected at the same time points to eliminate environmental interference caused by outdoor weather fluctuations. Even after rigorous homogenization screening, the inherent biological heterogeneity of individual seedlings could not be completely eliminated, which may have resulted in significant inter-group differences in the parameters measured on Day 0. Non-destructive physiological indicators (SPAD and electrophysiological indicators) were measured repeatedly on the same seedling at each sampling time point; for destructive sampling of biomass, separate replicate plants were used to avoid interference caused by the sampling procedure.

Despite careful morphological homogenization, significant inter-group differences at Day 0 may stem from hidden biological variation. Root-system characteristics, pre-sampling micro-environmental effects and internal nitrogen partitioning differ among visually similar seedlings, generating baseline divergence in SPAD and total nitrogen values. Subsequent SeMet-induced responses were evaluated based on temporal changes relative to each group’s initial condition, minimizing the influence of this baseline variation on treatment comparisons.

### 2.2. Experimental Treatment

#### 2.2.1. Adaptive Culture of *Cv*

Substrate culture (volume ratio of peat:vermiculite = 2:1) was used to carry out *Cv* adaptive culture experiments, and *Cv* seedlings were irrigated with a 1/2 concentration of Hoagland nutrient solution, formulated as: KNO_3_: 2.5 mM, NH_4_H_2_PO_4_: 1.0 mM, Ca(NO_3_)_2_·4H_2_O: 2.5 mM, MgSO_4_·7H_2_O: 1.0 mM, H_3_BO_3_: 23.1 µM, CuSO_4_·5 H_2_O: 0.16 µM, ZnSO_4_·7H_2_O: 0.38 µM, MnCl_2_·4H_2_O: 4.55 µM, Na_2_MoO_4_·2H_2_O: 0.19 µM, and Fe-EDTA: 50 µM. The processing period was 21 days [18].

#### 2.2.2. Different SeMet Selenium Enhancement Treatments

Root application was selected, and *Cv* seedlings at the 2–3 cotyledon stage were treated with different SeMet levels (analytical purity ≥ 98%): Ck—0 mg/L, S1—50 mg/L, S2—100 mg/L, S3—150 mg/L, S4—200 mg/L, S5—250 mg/L, and S6—300 mg/L SeMet. Each group was set with 3 biological replicates (n = 3) for 10 days [18]. During the treatment period, the pH of the nutrient solution remained stable, consistent with the experimental background conditions.

### 2.3. Growth Characteristics

#### 2.3.1. Determination of Total Chlorophyll and Total Nitrogen Content

According to method [19], we used a chlorophyll meter (0–99.9 SPAD, IN-YL03, Shandong Laiyin Photoelectric Technology Co., Ltd., Weifang, China) to measure total chlorophyll and total nitrogen content of *Cv* leaves. For SPAD determination, multiple measurement points on different mature leaves were recorded for each biological replicate, and the mean value was calculated to minimize measurement variability arising from leaf structural heterogeneity.

#### 2.3.2. Biomass

After harvest, following method [20], we collected the roots, stems, and leaves of *Cv* treated with different SeMet levels, and the fresh weight (FW, g/plant) was measured using a balance (AR124CN analytical balance (readability 0.0001 g), OHAUS Corporation, Shanghai, China).

### 2.4. Leaf Intrinsic Electrophysiological Characteristics

**Figure 1:**
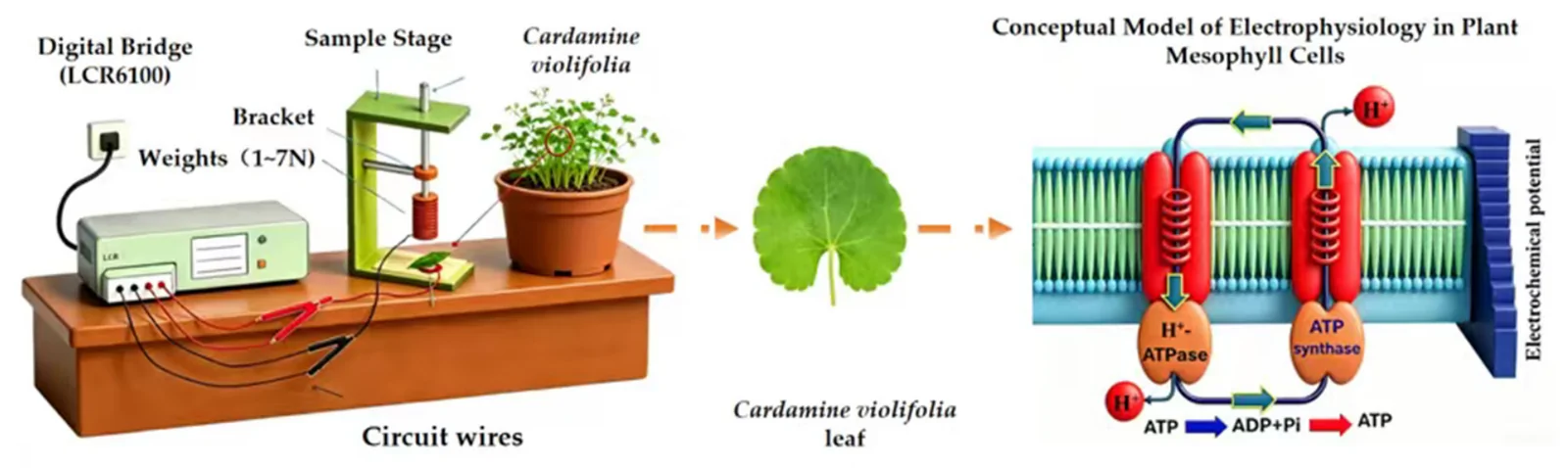
Diagram of a plant electrophysiology apparatus.

According to method [21], we measured the capacitance (Cp, capacitance), resistance (R, resistance), and impedance (Z, impedance) of the *Cv* leaves (Figure 1). We constructed the electrophysiological models with different clamping forces (F, force, F = 1–7 N, every 1N measured once) in Equations (1)–(4), where F is the clamping force, and the unit is N; M is the iron block, m is the total mass of the plastic rod of the electrode sheet, and the unit is kg. g is the acceleration of gravity, taking 9.8 N/kg; x_0_, y_0_ and p_0_ are the intercepts of Cp, R and Z, respectively. h, k_1_ and k_2_ are the independent variables of Cp, R and Z, respectively. b_1_ and b_2_ are the power exponent coefficients of Cp, R and Z, respectively.

(1) $$F=\left(M+m\right)g$$

(2) $$\mathrm{Cp}=x_{0}+\mathrm{hF}$$

(3) $$R=y_{0}+k_{1}e^{-b_{1}F}$$

(4) $$Z=p_{0}+k_{2}e^{-b_{2}F}$$

According to method [22], we determined the capacitive reactance (Xc) and the relationship between Xc and F, as shown in Equations (5) and (6). According to Z, R, and Xc of *Cv* leaves, we calculated the inductive reactance (XL) of *Cv* leaves and the change in XL in Equations (7) and (8), where π = 3.1416; U= 1.5 V; q_0_, k_3_, and b_3_ are the intercept, independent variable coefficient, and power exponent coefficient of Xc and F, respectively; and a_0_, k_4_, and b_4_ are the intercept, independent variable coefficient, and power exponent coefficient of XL and F, respectively.

(5) $$X_{c}=\frac{1}{2\pi \mathrm{fCp}}$$

(6) $$\mathrm{Xc}=q_{0}+k_{3}e^{-b_{3}F}$$

(7) $$\frac{1}{-X_{L}}=\frac{1}{Z}-\frac{1}{R}-\frac{1}{\mathrm{Xc}}$$

(8) $$X_{L}=a_{0}+k_{4}e^{-b_{4}F}$$

When F = 0, we derived the intrinsic capacitance (ICp), intrinsic resistance (IR), intrinsic capacitive reactance (IX_C_), intrinsic inductive reactance (IX_L_), and intrinsic impedance (IZ); see Equations (9)–(13).

(9) $$\mathrm{ICp}=\frac{1}{2\pi \mathrm{fI}X_{L}}$$

(10) $$\mathrm{IR}=y_{0}+k_{1}$$

(11) $$\mathrm{IXc}=q_{0}+k_{3}$$

(12) $$IX_{L}=a_{0}+k_{4}$$

(13) $$\frac{1}{\mathrm{IZ}}=\frac{1}{\mathrm{IR}}+\frac{1}{\mathrm{IXc}}-\frac{1}{IX_{L}}$$

### 2.5. Leaf Electrophysiological Water Metabolism

According to method [23], we calculated the leaf intracellular water-holding capacity (IWHC), effective thickness (d), intracellular water use efficiency (IWUE), intracellular water-holding time (IWHT), and water transfer rate (WTR); see Equations (14)–(18). Among them, U is 1.5 V; d is the specific effective thickness of *Cv* leaves; and h is the independent variable coefficient for Cp and F.

(14) $$\mathrm{IWHC}=\sqrt{\mathrm{IC}p^{3}}$$

(15) $$d=\frac{U^{2}h}{2}$$

(16) $$\mathrm{IWUE}=\frac{d}{\mathrm{IWHC}}$$

(17) IWHT=C×Z

(18) $$\mathrm{WTR}=\frac{\mathrm{IWHC}}{\mathrm{IWHT}}$$

### 2.6. Leaf Electrophysiological Nutrient Transport

According to method [24], we calculated the *Cv* leaf nutrient flux per unit area (UNF), nutrient translocation rate (NTR), nutrient translocation capacity (NTC), active transport flow of nutrient (UAF), and nutrient active translocation capacity (NAC); see Equations (19)–(23), respectively.

(19) $$\mathrm{UNF}=\frac{R}{\mathrm{IXc}}+\frac{R}{IX_{L}}$$

(20) $$\mathrm{NTR}=\frac{\mathrm{IWHC}}{\mathrm{IWHT}}$$

(21) NTC=UNF×NTR

(22) $$\mathrm{UAF}=\frac{IX_{C}}{IX_{L}}$$

(23) NAC=UAF×NTR

### 2.7. Leaf Electrophysiological Metabolic Activity

According to method [13], we calculated the leaf electrophysiological relative metabolic flux (MF), relative metabolic rate (MR), and relative metabolic activity (MA) in Equations (24)–(26).

(24) $$\mathrm{MF}=\frac{1}{\mathrm{IR}\times \mathrm{IZ}\times IX_{C}\times IX_{L}}$$

(25) MR=WRT×NAC

(26) $$\mathrm{MA}=\sqrt[{6}]{\mathrm{MF}\times \mathrm{MR}}$$

### 2.8. Selenium Fortification Based on Electrophysiological Water Metabolism and Nutrient Transport in Leaves

Referring to method [25], we calculated the selenium enhancement factors based on leaf electrophysiological water metabolism and nutrient transport characteristics, leaf electrophysiological selenium excretion capacity (ES1), selenium dilution capacity (ES2), selenium ultrafiltration capacity (ES3), selenium total transport capacity (STC), and selenium enhancement capacity (ESR) in Equations (27)–(31).

STC = UNF × WTR(27)

ES1 = 0.25 × (IR + IZ + IX_C_ + IX_L_) (28)

ES2 = 0.25 × (Icp + d + IWHC + STC)(29)

ES3 = 0.25 × (IWUE + IWHT + UNF + WTR)(30)

(31) $$\mathrm{ESR}=\frac{\text{ES1}+\text{ES2}+\text{ES3}}{3}$$

### 2.9. Statistical Analysis

SigmaPlot 15.0 was used to fit the electrophysiological equation of *Cv* leaves; SPSS 27.0 was used to analyze data differences and significance. Different letters (a–f) indicated significant differences, and the results are expressed as “mean ± standard deviation”. SPSS 21 was used to perform one-way analysis of variance (ANOVA) and the least significant difference test (LSD, *p* < 0.05), and Origin 2025 was used to finish plots.

## 3. Results

### 3.1. Growth Characteristics

To eliminate the influence of significant baseline differences among groups at Day 0, values measured at each sampling day were normalized to their corresponding Day-0 value to calculate relative proportional changes. This normalization approach was applied to evaluate the true regulatory effects of SeMet on seedling growth, independent of initial growth variation.

Table 1 and Table 2 show the relative proportional changes in total chlorophyll (SPAD value) content and total nitrogen (TN) content of *Cardamine violifolia* (*Cv*) seedlings over a 10-day period under different SeMet levels. The results showed that the SPAD and TN both reached their maximum on day 4, indicating that day 4 is the optimal time for SeMet application. Accordingly, growth traits and electrophysiological characteristics measured on day 4 were selected for further analysis.

**Figure 2:**
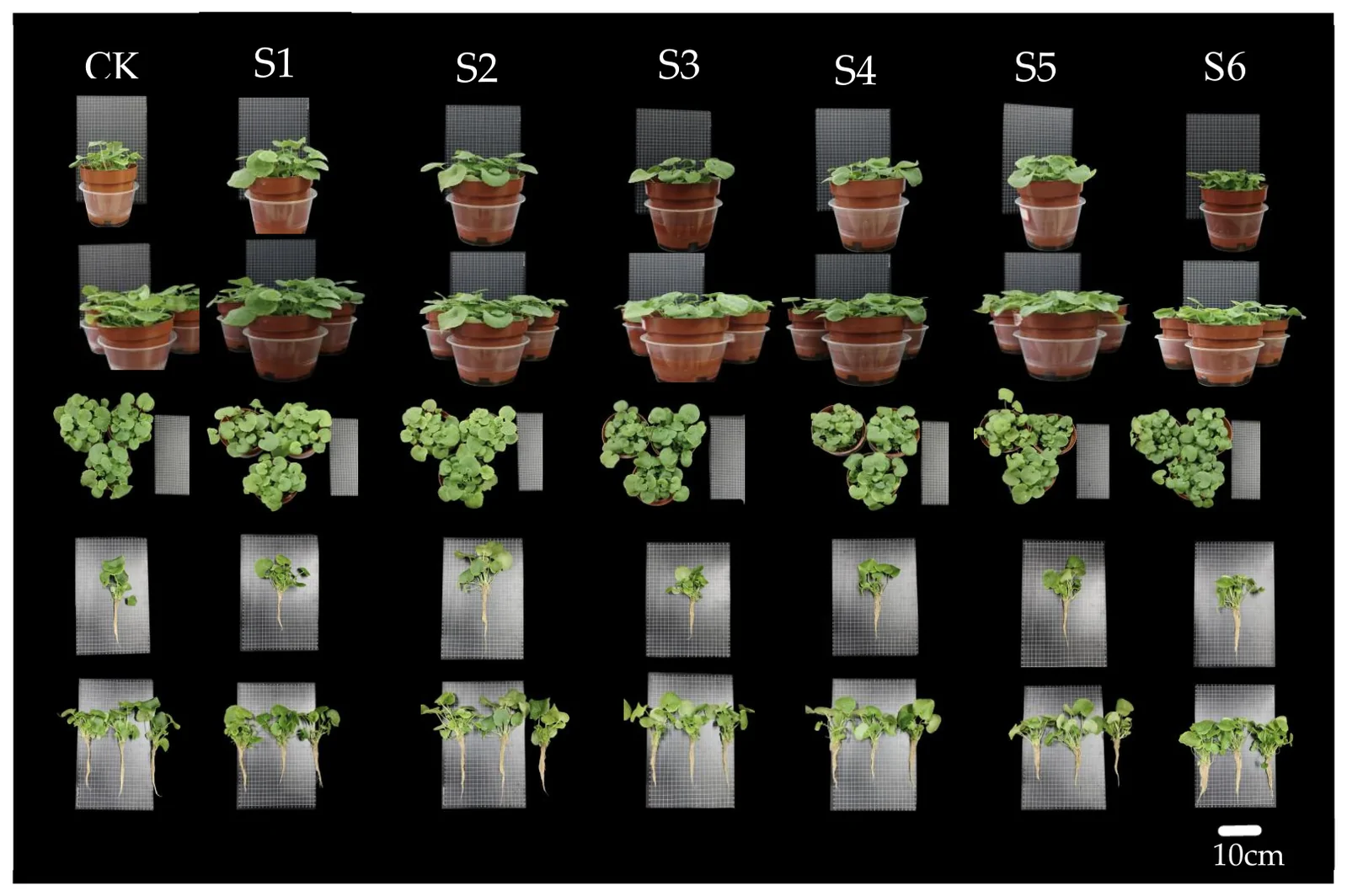
Morphological performance of *Cardamine violifolia* seedlings subjected to different SeMet concentrations at day 4. Images were obtained using three shooting modes: eye-level photography of potted plants, top-down canopy imaging, and flat-laid observation of thoroughly washed whole seedlings.

Table 3 shows that relative changes in root, stem, and leaf fresh weight differed substantially among SeMet levels at day 4. Compared to CK, only S2 promoted the fresh-weight of all organs. By contrast, S1 and S3–S6 inhibited fresh-weight accumulation in roots, stems, and leaves. Table 4 and Figure 2 show the plant height, leaf area, SPAD, TN and growth traits of *Cv* leaves at day 4. The results showed S2 exhibited the greatest growth traits, with increases of 18.91% and 23.96% compared with CK, which is consistent with its superior growth performance. Nevertheless, these growth traits cannot accurately distinguish the fine differences across other treaments (Table 3 and Table 4).

### 3.2. Leaf Electrophysiological Fitting Equation

**Figure 3:**
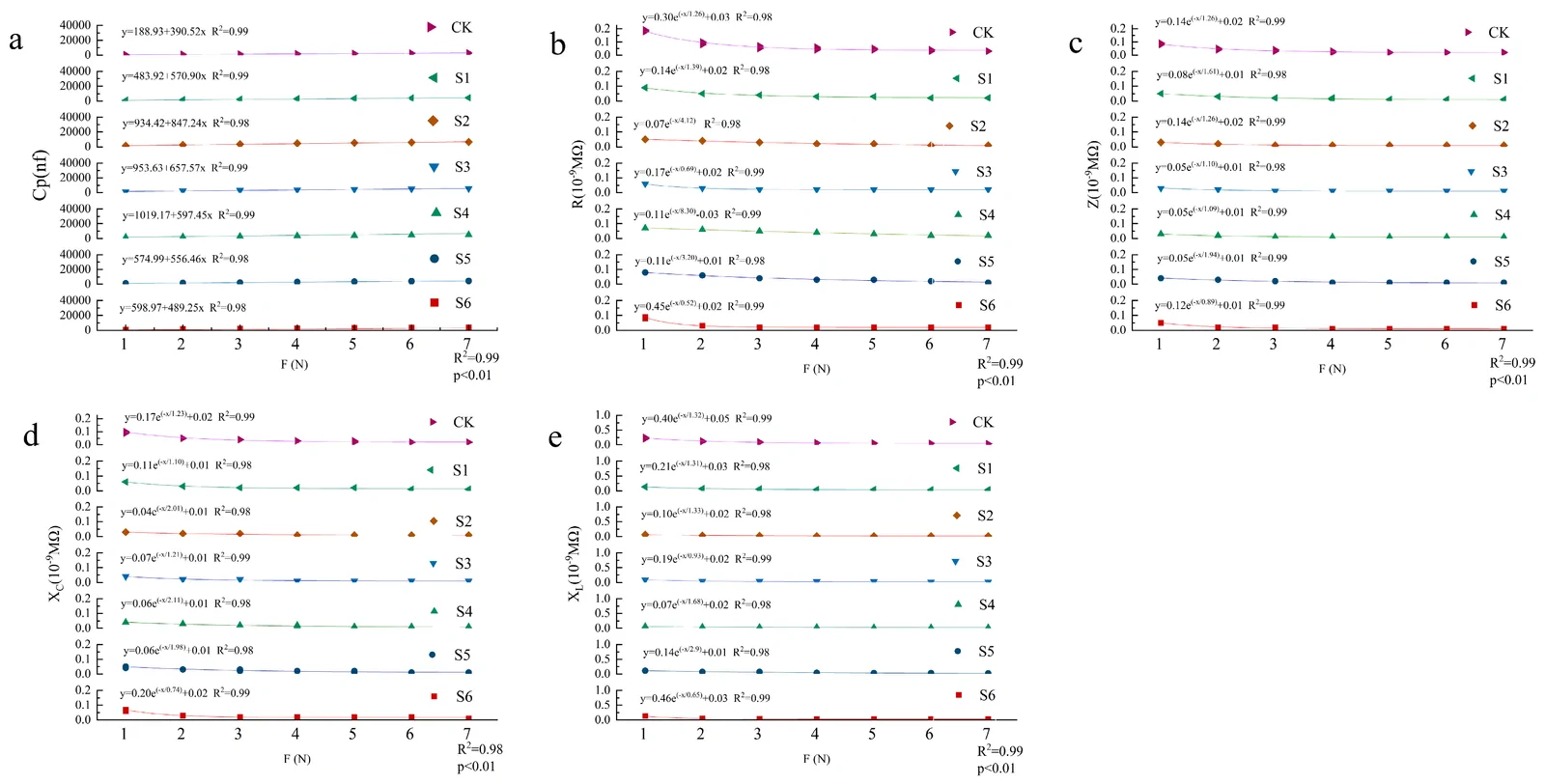
Electrophysiological equations for (**a**) Inherent capacitance, Cp; (**b**) inherent resistance, R; (**c**) inherent impedance, Z; (**d**) inherent capacitive reactance, Xc; (**e**) inherent inductive reactance, XL of *Cv* leaves under different SeMet conditions and for different clamping forces F (1–7 N), *p* < 0.01.

Figure 3 shows the electrophysiological equations of leaf capacitance (Cp, Figure 3a), resistance (R, Figure 3b), impedance (Z, Figure 3c), inductive reactance (X_C_, Figure 3d), and capacitive reactance (X_L_, Figure 3e) with different clamping forces F (1–7 N) under different SeMet levels. The results showed that the electrophysiological equations for Cp, R, Z, X_C_, and X_L_ were significantly correlated with F (R^2^ = 0.98–0.99), whereas all treatments (CK-S5) were significantly different in *Cv* leaves (*p* < 0.01).

### 3.3. Leaf Intrinsic Electrophysiological Parameters

**Figure 4:**
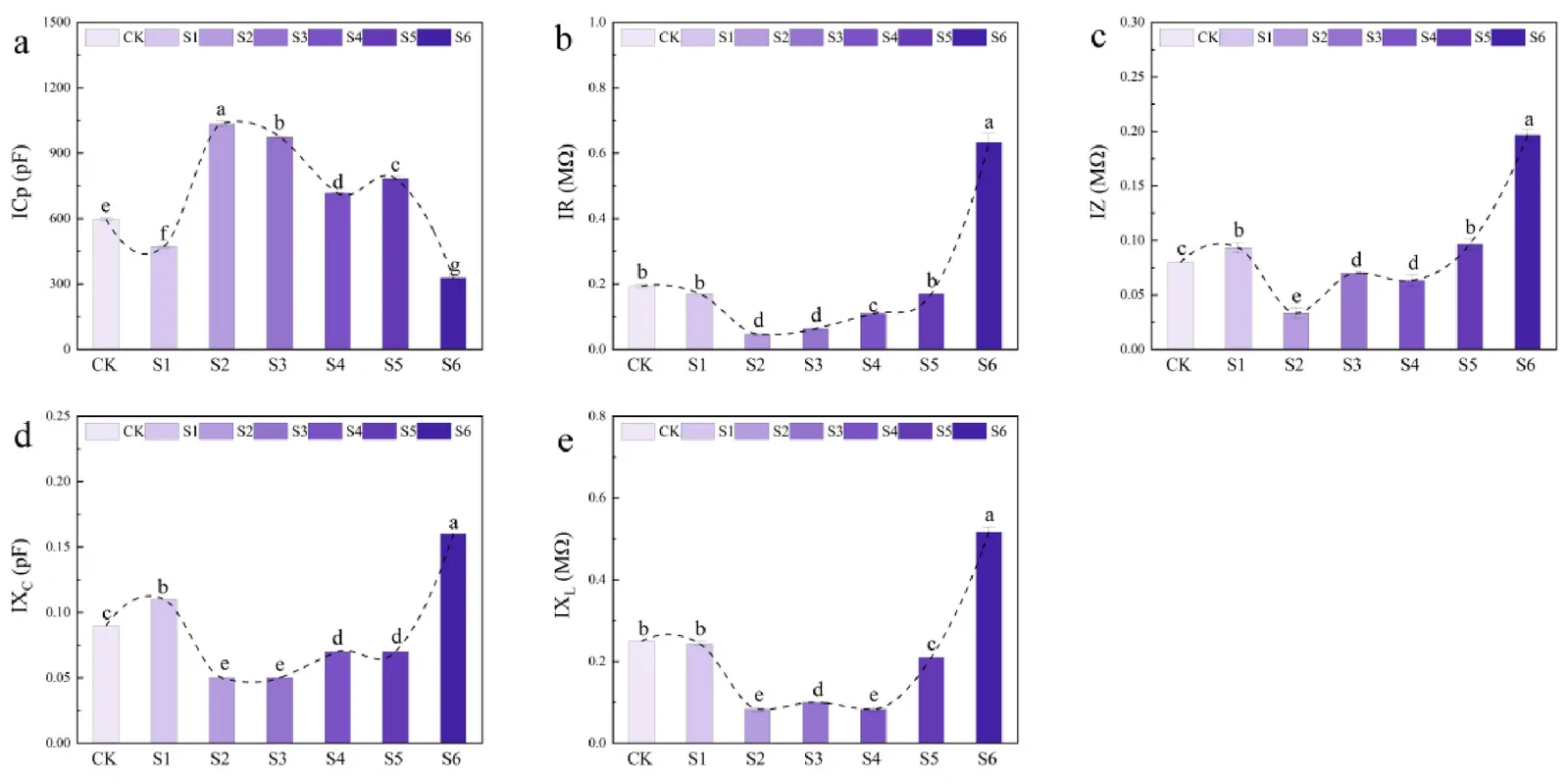
Intrinsic electrophysiological parameters of *Cv* leaves under different SeMet levels. Inherent capacitance, ICp (**a**); inherent resistance, IR (**b**); inherent impedance, IZ (**c**); inherent capacitive reactance, IXc (**d**); inherent inductive reactance, IX_L_ (**e**). Each value represents the mean ± standard deviation (n = 3). Different lowercase letters indicate significant differences between groups (*p* < 0.05).

Figure 4 represents *Cv* leaf intrinsic electrophysiological parameters, including ICp (Figure 4a), IR (Figure 4b), IZ (Figure 4c), IX_C_ (Figure 4d), and IX_L_ (Figure 4e). The results showed that S2-S5 promoted ICp, but inhibited IR, IZ, IX_C_, and IX_L_. Compared with CK, the ICp of S2 was increased by 1.73-fold, but the IR, IZ, IX_C_, and IX_L_ of S2 were increased by 73.68%, 62.5%, 44.4%, and 68%, respectively, compared with CK.

### 3.4. Leaf Electrophysiological Water Metabolism and Nutrient Transport

**Figure 5:**
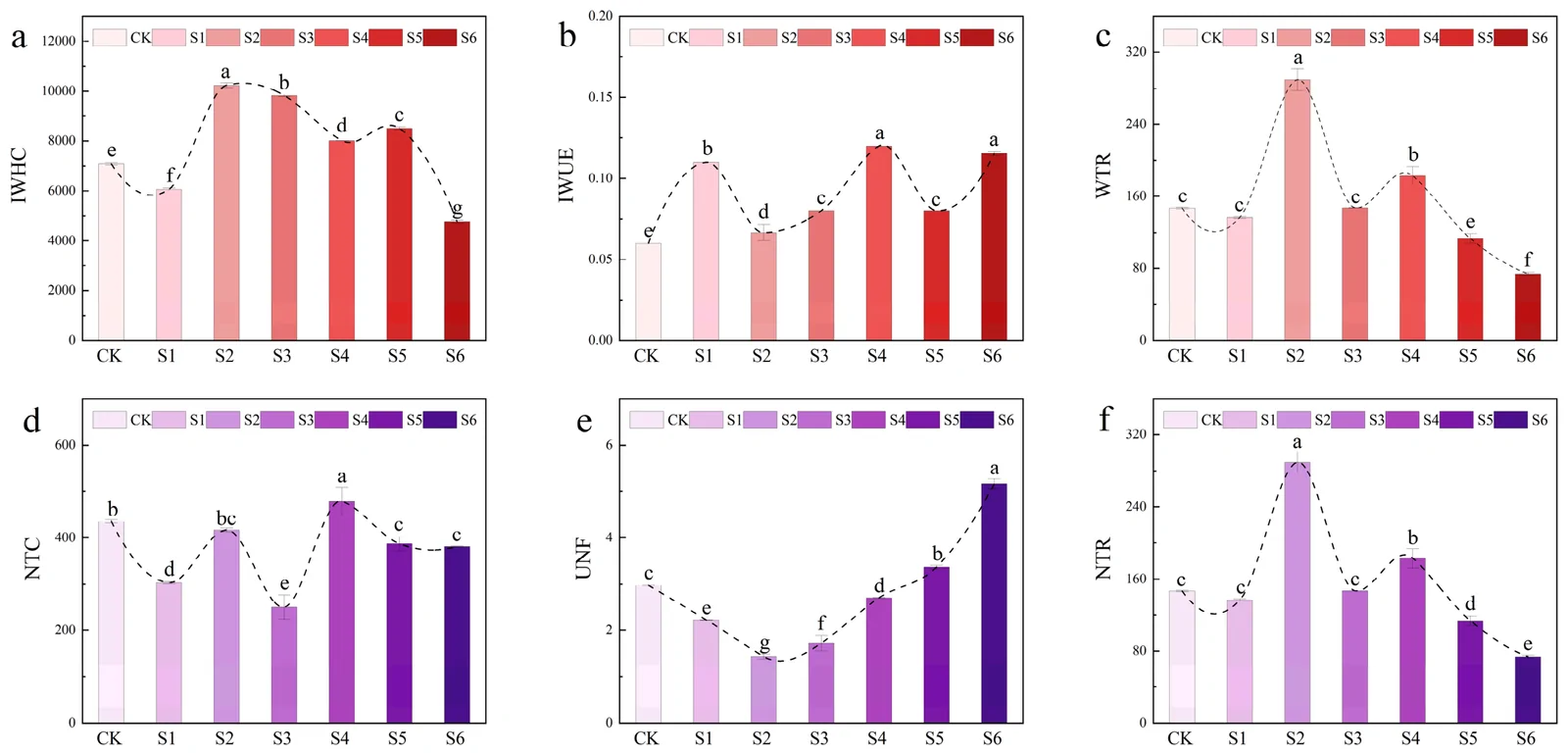
Intracellular electrophysiological water metabolism and nutrient transport of *Cv* leaves under different SeMet levels. (**a**) Intracellular water-holding capacity, IWHC; (**b**) intracellular water-holding time, IWUE; (**c**) water transfer rate, WTR; (**d**) unit nutrient transport capacity, NTC; (**e**) unit nutrient flux, UNF; (**f**) unit nutrient transport rate, NTR. Each value represents the mean ± standard deviation (n = 3). Different lowercase letters indicate significant differences between groups (*p* < 0.05).

Figure 5 shows the electrophysiological water metabolism and nutrient transport of *Cv* leaves under different SeMet. Figure 5a–c show the electrophysiological water metabolism of *Cv* leaves, including IWHC, IWUE, and WTR. Figure 5d–f show the electrophysiological nutrient transport of *Cv* leaves, including NTC, UNF, and NTR. With increasing SeMet levels, IWHC, IWUE, and WTR showed a sequential trend in electrophysiological water metabolism. The IWHC and WTR of S2 were the largest, at 1.44 and 1.93 times those of CK, respectively. The IWHC of S6 was the smallest, 32.8% lower than that of CK. In electrophysiological nutrient transport, NTC, UNF, and NTR showed a sequential trend. The NTR of S2 was the largest, 1.97-fold larger than CK. The UNF of S2 was the smallest, 50.17% lower than that of CK.

### 3.5. Leaf Electrophysiological Metabolic Activities

**Figure 6:**
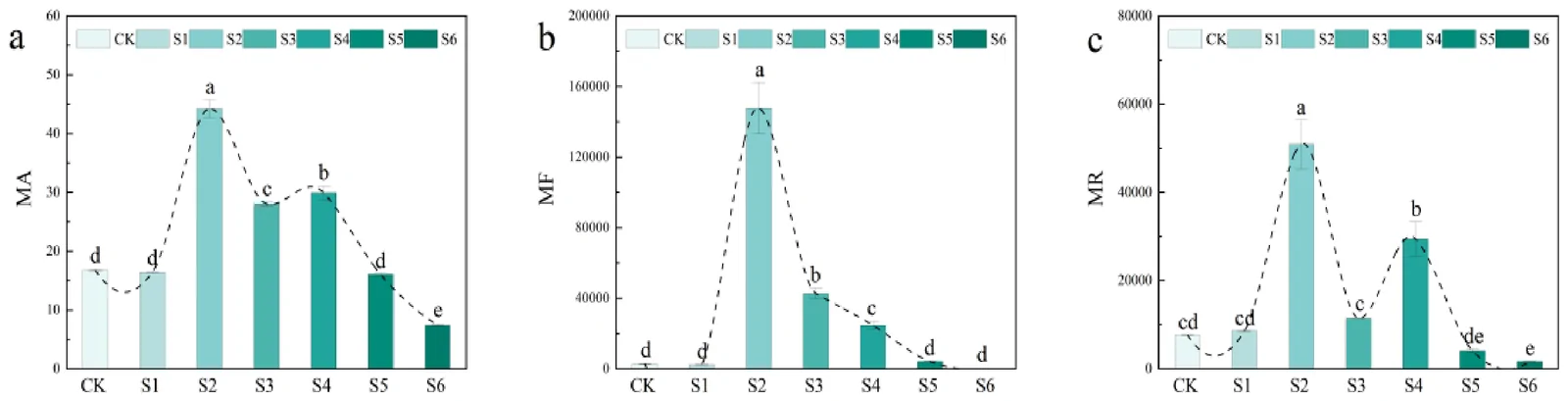
Electrophysiological metabolic activities of *Cv* leaves under different SeMet levels. (**a**) Metabolic activity, MA; (**b**) membrane fluidity, MF; (**c**) membrane resistance, MR. Each value represents the mean ± standard deviation (n = 3). Different lowercase letters indicate significant differences between groups (*p* < 0.05).

Figure 6 shows the electrophysiological and metabolic activities of *Cv* leaves under different SeMet levels. With SeMet increasing, MF, MR, and MA showed a sequential trend from CK to S6. The MF and MA of S2 were the largest, increasing by 51.27-fold and 2.64-fold compared to CK, respectively.

### 3.6. Electrophysiological Selenium Enhancement Characteristics of Cv Leaves

**Figure 7:**
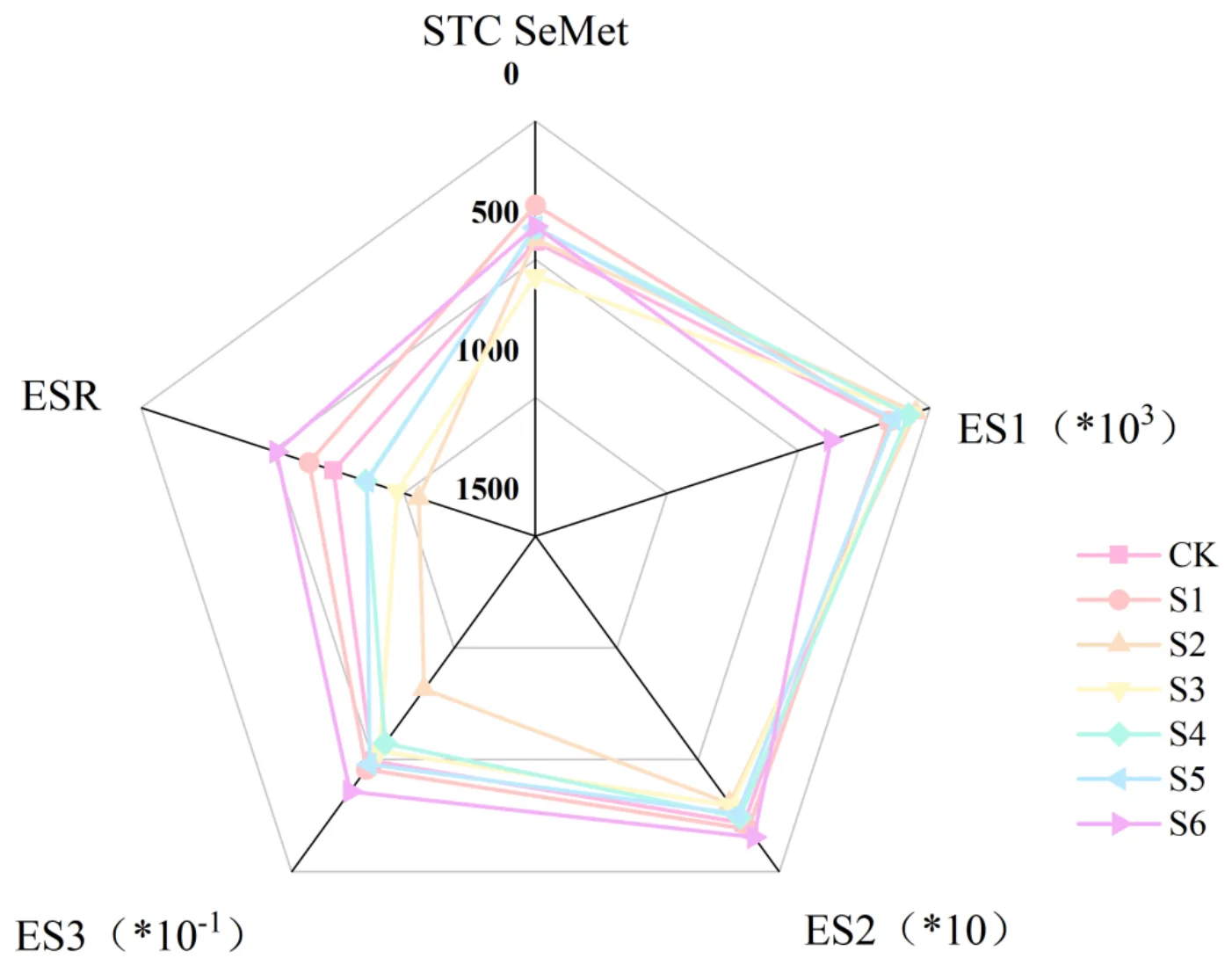
Electrophysiological selenium enhancement characteristics of *Cv* leaves. STC: SeMet transport capacity; ES1: electrophysiological selenium excretion capacity; ES2: selenium dilution capacity; ES3: selenium ultrafiltration capacity; ESR: electrophysiological selenium enhancement characteristic factor. Each value represents the mean ± standard deviation (n = 3).

Figure 7 shows the electrophysiological selenium enhancement characteristics of *Cv* leaves under different SeMet levels. The results showed that ESR in S2 was the highest, increasing by 29.06% compared to CK, with increases of 28.15% (STC SeMet), 2.24% (ES1), 40.42% (ES2), and 64.70% (ES3).

### 3.7. Correlation Analysis

**Figure 8:**
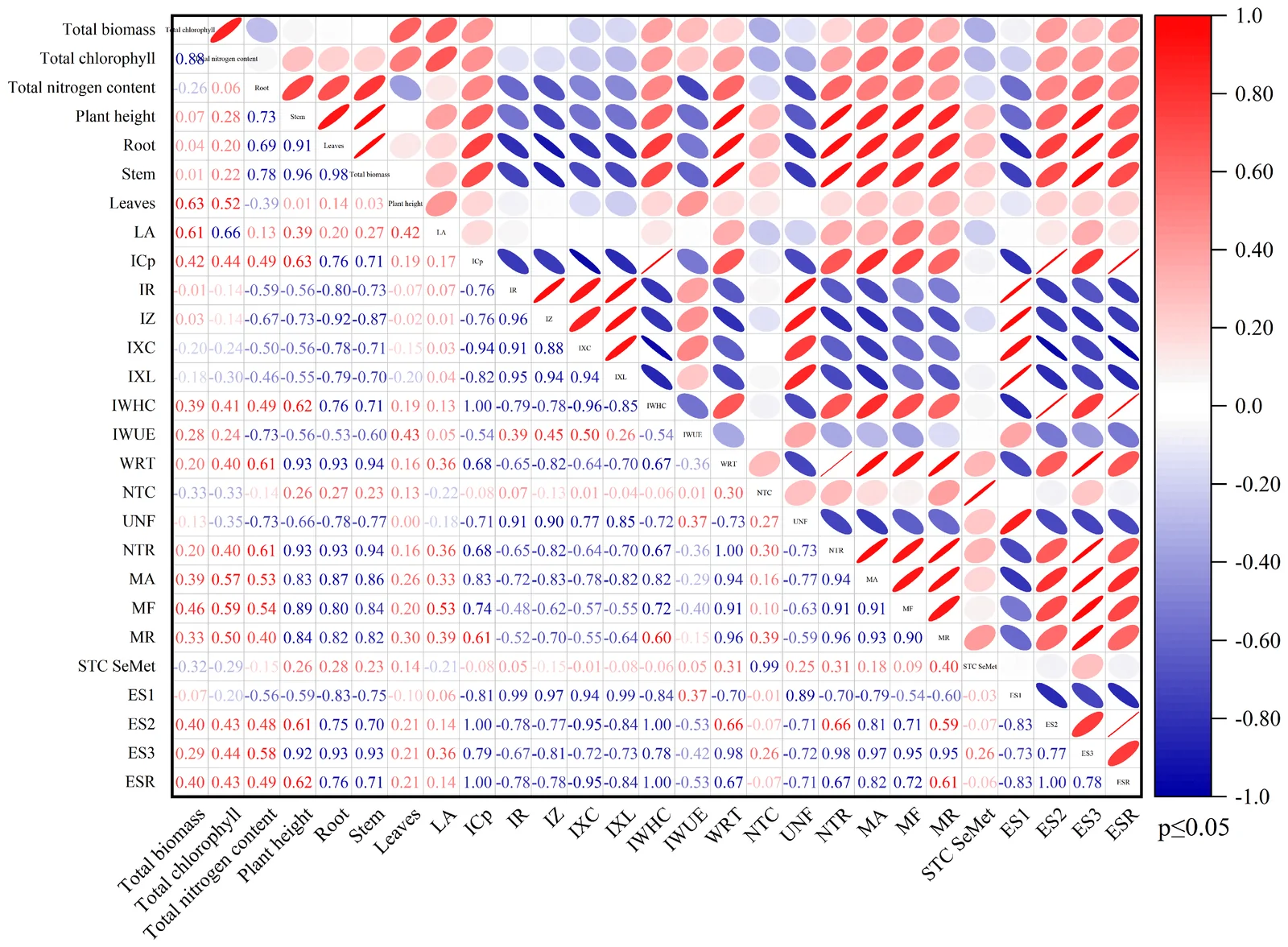
Correlation analysis based on the growth traits and electrophysiological characteristics of *Cv* leaves under different SeMet conditions (*p* ≤ 0.05). All characteristics are defined in the list of abbreviations.

Figure 8 presents a correlation analysis based on growth traits and electrophysiological characteristics of *Cv* leaves under different SeMet levels. It showed ESR was significantly positively correlated with total biomass (R^2^ = 0.89, *p* ≤ 0.01) and leaf area (R^2^ = 0.85, *p* ≤ 0.05). S2 had the highest ESR, consistent with the maximum biomass and chlorophyll content. Conversely, ES1 was significantly elevated in the S4–S6 treatments. ES2 was positively correlated with plant height (R^2^ = 0.78, *p* ≤ 0.05) and total nitrogen content (R^2^ = 0.82, *p* ≤ 0.05). Otherwise, there was a negative correlation between ES3 and root biomass (r = −0.74, *p* ≤ 0.05).

## 4. Discussion

### 4.1. Limitations of Growth Traits to Characterize Selenium Biofortification Capacity of Cv

Based on the growth traits of *Cv*, we found that the plant height, leaf area, total biomass, and chlorophyll content (Table 3 and Table 4) of *Cv* were highest on day 4, indicating that SeMet enhances carbon assimilation capacity by promoting the synthesis of photosynthetic pigment (chlorophyll) to enhance the growth of *Cv* [25]. Moreover, we found growth traits to characterize the selenium biofortification capacity of *Cv*. In this study, compared with S2, S3-S6 showed no significant difference in chlorophyll and total nitrogen content (Table 1 and Table 2). However, root, stem, and leaf biomass decreased significantly (Table 3) due to an accumulation of reactive oxygen induced by high SeMet levels. However, chlorophyll levels were maintained in the short term, and high SeMet levels inhibited dry matter accumulation in *Cv* [23]. In addition, this study confirms the decoupling of *Cv* growth and physiological metabolism, demonstrating asynchronous changes between biomass and physiological metabolism under different SeMet levels. These results are attributed to endogenous regulation of growth [18], energy allocation strategies [13], and environmental interactions of *Cv*, rather than to biomass accumulation alone [26]. Consequently, on day 4, there were no significant changes in the growth traits of S2 and S3, including no changes in biomass, plant height, leaf area, total chlorophyll content, or total nitrogen content, indicating that measuring growth traits alone cannot accurately reflect the SeMet formulations of *Cv*. To address this shortcoming, we further examined the electrophysiological water metabolism and nutrient transport of *Cv* leaves under different SeMet levels on day 4 (see Section 4.2).

### 4.2. Electrophysiological Water Metabolism, Nutrient Transport, and Metabolic Activities of Cv Leaves Under Different Semet Conditions

In this study, based on the electrical signals of *Cv* leaves, including leaf Cp, R, Z, Xc and X_L_ of *Cv*, an electrophysiological model was constructed to relate the above electrical signals to different gripping forces (Fs) [13] (R^2^ = 0.99, *p* < 0.05, Figure 3), and to quantify the water metabolism, nutrient transport, and metabolic activities of *Cv* leaves under different SeMet levels. It showed that the ICp of S2 increased significantly (Figure 4a), suggesting that vacuoles increased in volume at S2, which facilitated nutrient absorption in the cells [27]. Moreover, the IR and IZ of S2 decreased (Figure 4b–c), which facilitated the transport of SeMet and also the uptake of nutrients, such as K^+^ and NO_3_^−^ [9]. This result is consistent with the significant increase in SPAD (Table 1) and TN (Table 2) in S2. Importantly, we found that when the SeMet level increased, the IWHC (Figure 5a) and WTR (Figure 5c) of S3–S6 were significantly reduced, resulting in a reduction in active nutrient transport capacity (NTR, Figure 5f). At the same time, the IWUE (Figure 5b) of *Cv* increased significantly, leading to an increase in its passive transport capacity (NTC and UNF, Figure 5d,e). It has been shown that high SeMet levels induced membrane lipid peroxidation, leading to the inhibition of aquaporin (AQP) activity [28], water transport, and nutrient transport in plant species [27], thereby inhibiting the growth of *Cv*. Furthermore, we found that MA, MF and MR increased significantly in S2, whilst MA, MF and MR decreased significantly in S3–S6 (Figure 6). This indicated that S2 had the highest metabolic activities of *Cv*. However, with the increase in SeMet levels, the MA, MF and MR of S3–S6 decreased, which was attributed to high SeMet levels inhibiting the electrophysiologically active nutrient transport of *Cv*, leading to a reduction in nutrient store capacity in the vacuole [29]. In summary, S2 is the optimal SeMet level for *Cv*.

### 4.3. Establishment of Electrophysiological Selenium Enhancement Characteristics of Cv

Based on the electrophysiological water metabolism, nutrient transport, and metabolic activity of *Cv* leaves, we established the electrophysiological selenium enhancement characteristics and constructed five electrophysiological factors (Figure 7), including: electrophysiological efflux capacity (ES1), dilution capacity (ES2), ultrafiltration capacity (ES3), total SeMet transport capacity (STC), and enhancement capacity (ESR). In particular, ES1 represents the efficiency of transmembrane SeMet efflux, and it is negatively correlated with IR and IZ. ES2 represents the ability of SeMet to dilute and compartmentalize within cells, and it is significantly positively correlated with ICp and IWHC. ES3 represents the ultrafiltration retention capacity of the cell wall or vacuole of SeMet, and it is significantly negatively correlated with IXc. STC shows a significant positive correlation with electrophysiological nutrient transport; ESR is the weighted average of E1–E3, and it is used to characterize electrophysiological selenium enhancement characteristics of *Cv*. Correlation analysis shows (Figure 8) that ESR was significantly positively correlated with total biomass (R^2^ = 0.89, *p* ≤ 0.01) and leaf area (R^2^ = 0.85, *p* ≤ 0.05). S2 had the highest ESR, consistent with the maximum biomass and SPAD. Conversely, ES1 was significantly elevated in S4–S6, suggesting that high SeMet levels may induce a forced selenium-excreting metabolic pathway of *Cv* [30]. IR, IZ, IX_C,_ and IX_L_ of S6 increased, suggesting that high SeMet levels caused membrane lipid peroxidation and water metabolism reduction [22], leading to the biomass of S6 dropping to the lowest level. Furthermore, ES2 was positively correlated with plant height (R^2^ = 0.78, *p* ≤ 0.05) and TN (R^2^ = 0.82, *p* ≤ 0.05). This indicated that the diluting capacity promoted nitrogen metabolic homeostasis, thereby alleviating high SeMet stress [31]. Otherwise, we found a negative correlation between ES3 and the root biomass of *Cv* (R^2^ = −0.74, *p* ≤ 0.05). This suggests that high SeMet ultrafiltration capacity may inhibit SeMet transport from the roots to the aboveground parts, thereby limiting the efficiency of SeMet fortification [32]. In summary, the electrophysiological selenium enhancement characteristics can clearly distinguish the differential SeMet biofortification of *Cv*.

### 4.4. The Coupling Between Growth Traits and Electrophysiological Selenium Enhancement Characteristics

Based on a correlation analysis, we revealed the relationship between the growth traits and electrophysiological selenium enhancement characteristics of *Cv* (Figure 8). The results showed that ICp was significantly positively correlated with SPAD and TN (*p* ≤ 0.05), indicating that the cell membrane capacitance is consistent with the photosynthetic function and IR and IZ are negatively correlated with biomass, indicating that low resistance and impedance are conducive to dry matter accumulation [33]. Moreover, we found that electrophysiological parameters responded earlier than growth traits under various SeMet levels. For example, the ICp and IR of S3 showed more significant differences than those of S2 (Figure 5), However, the changes between S2 and S3 were not significant in terms of growth traits (Table 3), indicating that electrophysiological responses more sensitively captured “masked potentiating effects” under various SeMet levels [34], manifested by a significant promotion of electrophysiological responses in S2, as well as enhanced nutrient active transport capacity and metabolic activities in S2. Furthermore, based on electrophysiological water metabolism and nutrient transport of *Cv* leaves, we found that SeMet may regulate the expression of aquaporins and nutrient ion channels on the membrane, thereby modulating carbon–nitrogen coupling metabolism in *Cv*. In this study, S2 was the optimal SeMet level of *Cv*, exhibiting the highest biomass and ICp, indicating S2 prioritized the allocation of energy to growth metabolism, resulting in a higher SeMet enrichment efficiency of *Cv*. However, we found decoupling in S6, with growth traits showing a high SPAD value but low biomass, indicating that at high SeMet levels, *Cv* prioritizes the allocation of energy to resist growth stress rather than to support its growth [35]. Consequently, S6 reduces energy expenditure on growth, as indicated by the lowest ICp, IWHC, WTR, and NTR in S6 (high SeMet level). The above results indicate that both growth traits and electrophysiological responses showed high consistency. Hence, ESR could be an electrophysiological indicator to reflect selenium biofortification among plant species.

## 5. Conclusions

In this study, based on growth traits and electrophysiological characteristics, we found that day 4 was the optimal time, and S2 (100 mg/L) was the optimal level of SeMet utilization. Compared with growth traits, electrophysiological parameters were more sensitive to the selenium biofortification of *Cv*. This was manifested by the greatest change in ICp in S2, whilst IR, IZ, IX_C_, and IX_L_ showed opposite trends to ICp. Moreover, the increases in IWHC, WTR, and NTR most strongly promoted MA, thereby enhancing the highest SeMet fortification in S2. However, S3–S6 (>100 mg/L SeMet) inhibited IWHC and NTR in *Cv*, even though the ESR of S6 was higher than that of CK, while ES2 enhanced STC and ES1 in S6, which was still attributed to greater energy expenditure in counteracting high SeMet stress, leading to growth restriction in *Cv* at high SeMet levels. Correlation analysis showed ESR was significantly positively correlated with biomass, leaf area, and electrophysiological metabolic activity, suggesting that ESR could be an electrophysiological factor for monitoring selenium biofortification. Hence, plant electrophysiological technology could serve as a new method for analyzing selenium fortification. It holds considerable potential for smart agricultural monitoring, including precise applications and selenium tolerance among selenium hyperaccumulators.

## Figures and Tables

**Table 1:** Total chlorophyll content (SPAD) of *Cv* leaves over time under different SeMet levels. (Data in the table represent the relative proportional change compared with Day 0).

Treatment Days	0	2	4	6	8	10
CK	-	0	3.22%	4.60%	0	−10.29%
S1	-	26.40%	28.08%	30.11%	21.91%	22.01%
S2	-	40.42%	48.35%	44.46%	42.64%	41.20%
S3	-	27.31%	38.08%	33.95%	29.02%	27.93%
S4	-	33.55%	38.77%	38.39%	35.46%	33.93%
S5	-	14.24%	17.46%	17.78%	13.50%	12.54%
S6	-	25.22%	34.25%	31.89%	24.87%	23.55%

**Table 2:** Total nitrogen content of *Cv* leaves over time under different SeMet levels. (Data in the table represent the relative proportional change compared with Day 0).

Treatment Days	0	2	4	6	8	10
CK	-	0	12.37%	−1.37%	0	5.09%
S1	-	13.71%	35.39%	35.99%	14.82%	15.68%
S2	-	27.17%	57.64%	49.13%	34.49%	31.75%
S3	-	24.09%	44.65%	34.79%	14.32%	12.05%
S4	-	28.74%	46.60%	43.25%	22.67%	18.50%
S5	-	12.93%	20.21%	29.91%	7.52%	2.43%
S6	-	21.68%	41.13%	40.27%	10.80%	4.54%

**Table 3:** Root, stem, leaf and total biomass of *Cv* seedings under different SeMet treatments. Each value represents the mean ± standard deviation (n = 3). Different lowercase letters indicate significant differences between groups (*p* < 0.05).

Treatment	Root (Fw, g/plan)	Stem (Fw, g/plan)	Leaves (Fw, g/plan)	Total Biomass (Fw, g/plan)
CK	-	-	-	-
S1	−25%	−12%	−10%	−12%
S2	3%	33%	33%	30%
S3	−9%	−9%	−5%	−7%
S4	−44%	−8%	2%	−6%
S5	−47%	−14%	−5%	−13%
S6	−57%	−24%	−41%	−36%

**Table 4:** Plant height, leaf area, total chlorophyll content and total nitrogen content of *Cv* under different SeMet treatments. Each value represents the mean ± standard deviation (n = 3). Different lowercase letters indicate significant differences between groups (*p* < 0.05).

Treatment	Plant Height (cm)	LA (cm^3^)	Chlorophyll (SPAD)	Totalnitrogen (mg/g)
CK	-	-	-	-
S1	3.13%	9.13%	11.84%	13.67%
S2	4.06%	16.54%	18.91%	23.96%
S3	2.32%	7.14%	18.60%	23.53%
S4	4.04%	2.52%	13.40%	16.33%
S5	4.06%	3.20%	13.80%	6.98%
S6	3.31%	11.02%	17.23%	18.49%

## Data Availability

The original contributions presented in this study are included in the article. Further inquiries can be directed to the corresponding author.

## Abbreviations

The following abbreviations are used in this manuscript.

*Cv*	*Cardamine violifolia*
SeMet	selenomethionine
SPAD	total chlorophyll content
TN	total nitrogen content
F	clamping force
Cp	capacitance
R	resistance
Z	impedance
X_C_	capacitive reactance
X_L_	inductive reactance
ICp	intrinsic capacitance
IR	intrinsic resistance
IX_C_	intrinsic capacitive reactance
IX_L_	intrinsic inductive reactance
IZ	intrinsic impedance
IWHC	intracellular water-holding capacity
d	effective thickness
IWUE	intracellular water use efficiency
IWHT	intracellular water-holding time
WTR	water transfer rate
NTC	nutrient translocation capacity
UNF	nutrient flux per unit area
NTR	nutrient translocation rate
MA	metabolic Activity
MF	membrane Fluidity
MR	membrane Resistance
STC	SeMet transport capacity
ES1	electrophysiological selenium excretion capacity
ES2	selenium dilution capacity
ES3	selenium ultrafiltration capacity
ESR	electrophysiological selenium biofortification factor
